# Self-reported factors associated with community ambulation after stroke: The Canadian Longitudinal Study on Aging

**DOI:** 10.1371/journal.pone.0299569

**Published:** 2024-03-28

**Authors:** Ruth Barclay, Sandra C. Webber, Jacquie Ripat, Scott Nowicki, Robert Tate

**Affiliations:** 1 Department of Physical Therapy, College of Rehabilitation Sciences, Rady Faculty of Health Sciences, University of Manitoba, Winnipeg, Manitoba, Canada; 2 Department of Occupational Therapy, College of Rehabilitation Sciences, Rady Faculty of Health Sciences, University of Manitoba, Winnipeg, Manitoba, Canada; 3 Department of Community Health Sciences, Max Rady College of Medicine, Rady Faculty of Health Sciences, University of Manitoba, Winnipeg, Manitoba, Canada; International Institute of Health Management Research - New Delhi, INDIA

## Abstract

Community ambulation is frequently limited for people with stroke. It is, however, considered important to people with stroke. The objectives were to identify factors associated with self-reported community ambulation in Canadians aged 45+ with stroke and to identify factors associated with community ambulation specific to Canadian males and to Canadian females with stroke. Data were utilized from the Canadian Longitudinal Study on Aging Tracking Cohort. Multivariate logistic regression models were developed for community ambulation. Mean age was 68 (SE 0.5) years (45% female). In the final community ambulation model (n = 855), factors associated with being less likely to ‘walk outdoors sometimes or often’ included difficulty or being unable to walk 2–3 blocks (decreased endurance) vs. no difficulty. Being more likely to walk outdoors was associated with ‘better weather’ months and being 55–64 years of age vs 75–85. Differences were noted between the models of only males and only females. Decreased walking endurance is associated with a decreased likelihood of walking in the community—a factor that can be addressed by rehabilitation professionals and in community based programs.

## Introduction

Over 101 million people worldwide are living with stroke [1], including an estimated 878,500 Canadians [2]. After stroke, community ambulation is frequently limited [3] but it is considered essential or very important to people post stroke [4]. Community ambulation is defined as “independent mobility outside the home, which includes the ability to confidently negotiate uneven terrain, private venues, shopping centers and other public venues” [4]. This includes walking indoors and outdoors, outside of an individual’s home (e.g., a friend’s house, park, shopping mall). Walking in indoor and outdoor community environments is important, as it enables social participation in meaningful community activities [5].

There are many health benefits to community walking, however, many community dwelling people with stroke do not frequently walk in the community and engage in low levels of physical activity [6, 7]. It is estimated that at three months post stroke, approximately 37% of people are independent in community ambulation [8].

Numerous factors have been associated with being able to ambulate in the community after stroke, such as: higher gait speed [9–13], better endurance [3, 9, 14, 15], strength [15], self-efficacy related to balance or falls [9–12], balance [9–12], use of an assistive walking aid [9–12], and adequate lower limb motor function [9–12]. It has been suggested that after stroke, a minimum gait speed of 0.8 metres/second is required for community ambulation [16]. Younger age post stroke and absence of depression have been associated with being able to ambulate in the community [17], while positive health perceptions have been associated with community ambulation post stroke [18]. Poor weather is another challenge for individuals after stroke to walk outdoors in the community [6].

The Canadian Longitudinal Study on Aging (CLSA) provides the opportunity of studying a large number of self-reported factors potentially associated with community ambulation in a large Canadian sample [19]. The CLSA data provides an opportunity to understand multiple factors that limit or promote community ambulation in Canadians with stroke, which is important, as people with stroke frequently have goals of improving ambulation in the community. Looking at multiple self-reported factors specifically could be beneficial for future large studies or when in-person follow-up is not possible.

The **aim** of this study was to identify factors associated with community ambulation after stroke using data from a large population-based study of older adults.

### Objectives

1. To identify factors associated with community ambulation in Canadians aged 45+ with stroke.
2. To identify factors associated with community ambulation specific to Canadian males and to Canadian females with stroke.

## Materials and methods

### Data source

The Canadian Longitudinal Study of Aging (CLSA) is a large, population-based longitudinal study with a stratified random sample that is following more than 50,000 Canadians aged 45–85 at study baseline [20, 21]. There are two cohorts in the CLSA study: the Tracking cohort, a telephone interview, and the Comprehensive cohort, which includes site visits with physical assessments. At recruitment, individuals in long-term care, not able to speak in English or French, and those with cognitive impairments were excluded, as well as Canadian Forces members who are full-time, those living on federal First Nation reserves and First Nation settlements and those in the three Canadian Territories [20, 21]. The study received formal approval from the Health Research Ethics Board, University of Manitoba.

### Study sample

The CLSA Baseline Tracking dataset v 3.2 was used for this analysis (n = 21,171). We used data collected via telephone interview at baseline and the maintaining contact questionnaire (MCQ), which asked different, additional questions within the next 12–18 months [20, 21]. All variables were self-reported. The data was collected beween 2011 and 2016 [22].

#### Stroke sub sample

During the telephone baseline interview, each participant was asked the following questions: “1. Has a doctor ever told you that you have experienced a stroke or CVA (cerebrovascular accident)? 2. Has a doctor ever told you that you have experienced a mini-stroke or TIA? (Transient Ischemic Attack)? 3. Has a doctor ever told you that you suffer from the effects of a stroke, CVA (cerebrovascular accident), ministroke or TIA (Transient Ischemic Attack)?” [23]. If the answer to any of the questions was ‘yes’, the participant was determined to have had a stroke for the purpose of this study.

### Outcomes

#### Primary outcome

The primary outcome was community ambulation. This was represented by the MCQ interview variable, “Over the past 7 days, how often did you take a walk outside your home or yard for any reason? For example, for pleasure or exercise, walking to work, walking the dog, etc.” The response options were “Never, Seldom (1 to 2 days), Sometimes (3 to 4 days), Often (5 to 7 days)” [24]. In this analysis, the responses were dichotomized to ‘Sometimes or Often’ and ‘Never or Seldom’. This item is part of the Physical Activity Scale for the Elderly [25].

#### Explanatory variables

Detailed information about the variables and questions used in the tracking cohort is available from the CLSA website study protocol [21] and baseline data collection report [26]. Age was categorised by 10 year age groups: 45–54, 55–64, 65–74, 75–85. To evaluate self-rated health, participants were asked if their health was excellent, very good, good, fair or poor. The depression variable used in this study had four response options regarding how often an individual felt depressed in the last week, ranging from rarely or never to all of the time.

There were 42 questions regarding chronic health conditions. The number of conditions were summed and presented as zero to six, or seven or more chronic health conditions. The number of chronic conditions counted did not include the stroke questions used to define those with stroke.

Two variables regarding the presence of pain and intensity of pain were combined and recoded as pain-free, mild, moderate or severe pain. The number of falls in the previous 12 months were expressed as 0,1, or 2 or more falls. Two falls in a year suggest a higher risk for falls [27].

Variables regarding physical functioning were included from scales tested for validity and reliability that have also been shown to be correlated with performance based measures [20, 21]. Regarding walking ability, individuals responded whether they could walk without help or if they could walk with help from a person or walking aid. These variables were combined and recoded as walking with the help of a person or walking aid, or walking without help. Individuals who were unable to walk at all were identified as missing for the analysis (n = 4). The variable used to reflect endurance asked if an individual had difficulty walking the distance of two to three blocks. Responses were coded as no difficulty, difficulty, or unable. Standing up from a chair reflects leg strength [28]. The variable refers to difficulty standing up from a chair. The three response options were dichotomized as unable to stand up or difficulty standing up, and no difficulty standing up, due to very few participants answering unable.

Weather conditions at assessment time were reflected by the month of interview at the time of answering the primary outcome question. Control variables included income, education, marital status, living in an urban or rural setting, and province in Canada.

### Analyses

Analyses were conducted using SAS software, Version 9.4 (SAS Institute Inc., Cary, NC, USA). Descriptive analyses were conducted for all variables using mean and standard deviation (SD) or frequency and percent (%). Univariate binary logistic regression analysis was utilized, with the outcome of community ambulation. For the initial model, each independent variable in each univariate model which was statistically significant at p≤ 0.05, was included in the multivariable binary logistic regression model of community ambulation. Separate models were also established for males and females.

The final model was developed by including variables from the initial model that had at least one statistically significant response option. All the control variables were also included, regardless of significance. For the models by sex, a variable was included if it had at least one significant item from the initial model. For each model, only data from participants with complete data were used.

We had planned to use ordinal logistic regression, however, the proportional odds assumption was violated, therefore, the community ambulation outcome variable was dichotomized. The cutpoints for the community ambulation outcome were selected based on reviewing all possible cutpoints. Trimmed weights were used for weighted frequencies and analytic weights were used for regression models.

## Results

**Fig 1:**
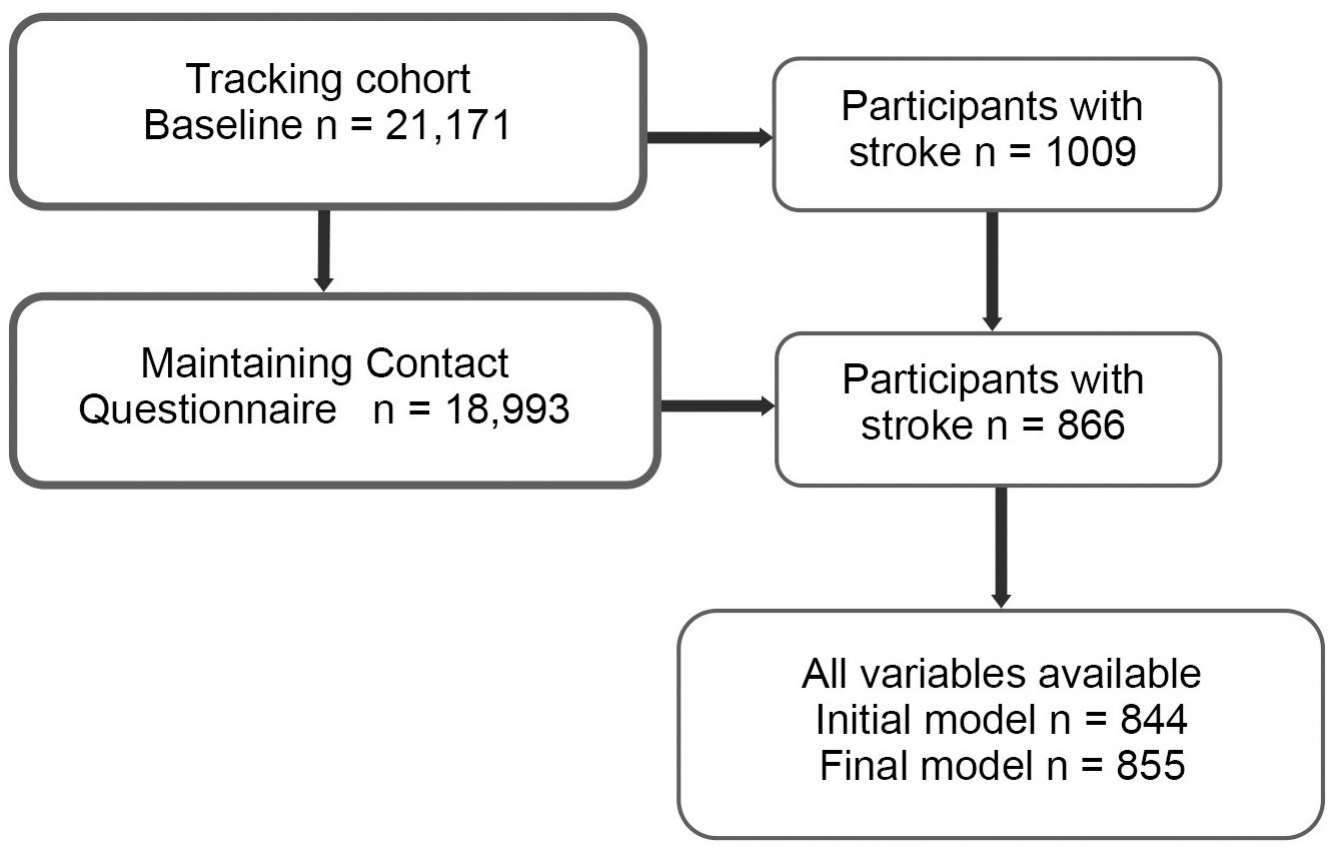
Study flow chart.

There were 21,171 participants in the tracking main wave, 1009 who had a stroke. The 1009 participants with stroke represent 515,206 (20,769) (weighted frequency (SE)) Canadians. 18,993 of the 21,171 participants completed the MCQ. Of the 1009 people with stroke, 866 had also answered the MCQ. See Fig 1 for the study flow chart. Participant characteristics are described in Table 1. Mean age was 68.3 (SE 0.5) years and 393 (45.4%) were female. Most participants rated their health as good, very good or excellent (n = 610, 70.4%), and 535 participants (61.8%) had pain that was classified as pain free or mild.

**Table 1:** Participant characteristics (non-weighted).

Category	Descriptor	Participants with stroke n = 866	
		n	%
**Walking outside the home or yard in the past 7 days**	never	218	25.2
	Seldom (1–2 days)	139	16.1
	Sometimes (3–4 days)	112	12.9
	Often (5–7 days)	396	45.7
	missing	1	0.1
**Sex**	female	393	45.4%
	male	473	54.6%
**Age**	Mean (SE)	68.3 (0.5)	
	45–54	69	8.0%
	55–64	180	20.8%
	65–74	248	28.6%
	75–85	369	42.6%
**Marital Status**	Single	58	6.7%
	Married / common law	511	59.0%
	Widowed	178	20.6%
	Divorced	91	10.5%
	Separated	28	3.2%
	missing	0	0.0%
**Living location**	Rural	161	18.6%
	Urban	705	81.4%
**Number of chronic conditions**	0/1	33	3.8%
	2	74	8.5%
	3	90	10.4%
	4	99	11.4%
	5	86	9.9%
	6	108	12.5%
	7+	376	43.4%
	e.g. hypertension, heart disease, osteoarthritis in the knee		
**General Health**	Poor	84	9.7%
	Fair	170	19.6%
	Good	299	34.5%
	Very good	227	26.2%
	Excellent	84	9.7%
	Missing	2	0.2%
**Depression**	All the time (5-7d/wk)	35	4.0%
	Occasionally (3-4d/wk)	76	8.8%
	Some of the time (1-2d/wk)	133	15.4%
	Rarely or never(<1)	621	71.7%
	Missing	1	0.1%
**Able to walk**	Walk with help of a person or used 1 or more mobility aids	286	33.0%
	Able to walk without help (no mobility aids or no help of a person)	576	66.5%
	Missing	4	0.5%
**Ability to walk 2–3 blocks (endurance)**	Unable	38	4.4%
	Difficulty	169	19.5%
	No difficulty	654	75.5%
	Missing	5	0.6%
**Pain**	Severe	82	9.5%
	Moderate	246	28.4%
	Mild	114	13.2%
	Pain free	421	48.6%
	Missing	3	0.3%
**Falls in past 12 months**	0	688	79.4%
	1	103	11.9%
	2+	70	8.1%
	missing	5	0.6%

All independent variables were statistically significant at p< = 0.05 in each univariate model, therefore, all 15 proposed explanatory and control variables were included in multivariate models for the initial model. 844 participants are represented in this model, 385 female and 459 male. Only the statistically significant variables (p< = 0.05) from this model were included in the final model. The final model includes 855 participants, 388 female and 466 male. The difference in sample size between the models is due to the number of people with complete data on all variables used in each model.

In the initial model, two items were associated with decreased odds of walking outside sometimes/often versus never/seldom: being unable to walk 2–3 blocks and having difficulty walking 2–3 blocks (decreased endurance), compared to no difficulty. See Table 2, which presents findings of the initial model and a description of each variable and category. Factors associated with increased odds of walking outside sometimes/often vs. never/seldom included: being in the 55–64 year age group compared to 75–85 years, walking in ‘better’ weather months compared to January, and having mild pain versus being pain-free. Differences were noted in the male and female specific models.

**Table 2:** Community ambulation—Initial model.

		All participants with stroke n = 844					Females with Stroke n = 385					Males with Stroke n = 459				
Variables	Descriptor	Odds Ratio	95% CI		F Value (df num, df den)	p	Odds Ratio	95% CI		F Value (df num, df den)	p	Odds Ratio	95% CI		F Value (df num, df den)	p
**General Health**	Poor vs Excellent	0.75	0.30	1.91	0.48 (4, 18663)	0.75	0.39	0.08	1.86	0.62 (4, 18663)	0.65	1.10	0.31	3.84	1.18 (4, 18663)	0.32
	Fair vs Excellent	0.77	0.35	1.68			0.73	0.19	2.81			0.57	0.20	1.58		
	Good vs Excellent	0.72	0.36	1.42			0.54	0.18	1.62			0.93	0.38	2.26		
	Very good vs Excellent	0.95	0.49	1.85			0.67	0.23	1.95			1.34	0.55	3.32		
**Depression**	All of the time (5-7days) vs Rarely or never (less than 1 day)	0.59	0.25	1.36	1.87 (3, 18664)	0.13	1.32	0.38	4.61	0.92 (3, 18664)	0.43	0.41	0.12	1.39	**3.13** (3, 18664)	**0.02**
	Occasionally (3–4 days) vs 4 Rarely or never (less than 1 day)	1.38	0.74	2.58			1.93	0.78	4.79			0.96	0.35	2.62		
	Some of the time (1–2 days) vs 4 Rarely or never (less than 1 day)	0.69	0.43	1.11			1.57	0.75	3.29			**0.35**	**0.18**	**0.72**		
**Chronic conditions**	0/1 vs. 7	2.44	0.94	6.37	1.18 (6, 18661)	0.31	3.08	0.53	18.08	0.66 (6, 18661)	0.68	3.44	0.94	12.53	1.52 (6, 18661)	0.17
	2 vs. 7	1.65	0.80	3.42			2.75	0.80	9.54			1.51	0.56	4.05		
	3 vs. 7	0.77	0.41	1.42			1.13	0.36	3.50			0.58	0.24	1.38		
	4 vs. 7	1.04	0.57	1.90			0.92	0.38	2.23			1.72	0.65	4.54		
	5 vs. 7	0.97	0.52	1.83			1.12	0.41	3.05			0.89	0.38	2.09		
	6 vs. 7	1.07	0.59	1.95			1.04	0.40	2.66			1.07	0.47	2.46		
**Able to walk**	Walk with help of person or aid vs Walk without help	0.79	0.52	1.20	1.15 (1, 18666)	0.28	0.84	0.43	1.62	0.28 (1, 18666)	0.60	0.77	0.44	1.36	0.80 (1, 18666)	0.37
**Endurance (Walk 2–3 blocks)**	Unable vs no difficulty	**0.17**	**0.07**	**0.41**	**11.74** (2, 18665)	**<0.0001**	**0.17**	**0.05**	**0.57**	**10.14** (2, 18665)	**< .0001**	**0.04**	**0.01**	**0.31**	**5.60** (2, 18665)	**0.004**
	Difficulty vs no difficulty	**0.38**	**0.24**	**0.61**			**0.22**	**0.11**	**0.45**			**0.44**	**0.22**	**0.88**		
**Leg strength (stand up)**	Unable to do or difficult vs no difficulty	1.14	0.77	1.71	0.43 (1, 18666)	0.51	0.79	0.43	1.46	0.57 (1, 18666)	0.45	1.37	0.76	2.46	1.10 (1, 18666)	0.29
**Pain**	Severe vs pain- free	0.80	0.44	1.47	2.32 (3, 18664)	0.07	0.60	0.22	1.65	**2.80** (3, 18664)	**0.04**	1.58	0.60	4.15	1.65 (3, 18664)	0.17
	Moderate vs pain- free	1.34	0.87	2.07			1.13	0.573	2.22			**2.12**	**1.07**	**4.18**		
	Mild vs pain- free	**1.78**	**1.02**	**3.11**			**2.55**	**1.15**	**5.65**			0.96	0.42	2.21		
**Falls in 12 months**	1 Fall vs 0 Falls	1.20	0.71	2.03	0.46 (2, 18665)	0.63	1.58	0.75	3.35	0.75 (2, 18665)	0.47	1.17	0.51	2.69	0.24 (2, 18665)	0.79
	2+ Falls vs 0 Falls	0.85	0.47	1.51			0.96	0.42	2.22			0.75	0.27	2.07		
**Weather (month)**	February vs January	2.47	0.98	6.25	1.56 (11, 18656)	0.10	3.04	0.74	12.49	**1.80** (11, 18656)	**0.05**	2.11	0.56	8.03	1.28 (11, 18656)	0.23
	March vs January	1.94	0.73	5.11			2.17	0.45	10.36			1.30	0.32	5.37		
	April vs January	**3.23**	**1.26**	**8.30**			3.71	0.78	17.61			1.65	0.45	6.10		
	May vs January	**2.95**	**1.33**	**6.58**			**3.85**	**1.08**	**13.68**			2.15	0.73	6.27		
	June vs January	**2.10**	**1.00**	**4.43**			**4.89**	**1.48**	**16.19**			0.72	0.27	1.95		
	July vs January	2.13	0.92	4.93			**7.52**	**2.03**	**27.86**			0.71	0.22	2.27		
	August vs January	1.99	0.83	4.75			3.60	0.98	13.20			1.17	0.31	4.33		
	September vs January	**3.69**	**1.38**	**9.87**			**8.69**	**2.21**	**34.18**			2.14	0.55	8.29		
	October vs January	2.58	0.91	7.31			**11.25**	**2.22**	**56.90**			1.12	0.31	4.01		
	November vs January	1.57	0.69	3.58			**3.67**	**1.00**	**13.52**			0.80	0.27	2.34		
	December vs January	1.14	0.52	2.50			1.56	0.46	5.28			0.63	0.22	1.77		
**Age group**	45–54 vs 75–85	1.11	0.58	2.10	1.76 (3, 18664)	0.15	0.57	0.18	1.77	**2.99** (3, 18664)	**0.03**	1.27	0.53	3.06	0.17 (3, 18664)	0.91
	55–64 vs 75–85	**1.75**	**1.07**	**2.86**			**2.47**	**1.20**	**5.12**			1.25	0.61	2.56		
	65–74 vs 75–85	1.23	0.81	1.86			1.08	0.53	2.20			1.03	0.57	1.85		
**Sex**	Female vs Male	0.69	0.47	1.01	3.67 (1, 18666)	0.06	-	-	-	-	-	-	-	-	-	-
**Income**	>$20,000, < $50,000 vs <20,000	0.73	0.40	1.32	0.64 (5, 18662)	0.67	0.89	0.39	2.01	1.23 (5, 18662)	0.29	0.61	0.19	1.94	0.74 (5, 18662)	0.59
	≥$50,000, < $100,000 vs < $20,000	0.70	0.36	1.37			1.38	0.52	3.70			0.39	0.11	1.32		
	≥$100,000, < $150,000 vs < $20,000	0.62	0.25	1.55			0.33	0.06	1.71			0.50	0.12	2.13		
	$150,000 + vs < $20,000	1.16	0.37	3.62			1.34	0.24	7.40			0.55	0.09	3.48		
	No response vs < $20,000	1.00	0.44	2.25			1.70	0.60	4.82			0.41	0.09	1.90		
**Education**	No post-secondary degree, certificate or diploma vs ≤ High school	0.97	0.50	1.88	0.99 (7, 18660)	0.43	1.70	0.57	5.15	0.43 (7, 18660)	0.89	0.75	0.30	1.90	1.24 (7, 18660)	0.28
	Trade certificate, diploma or apprenticeship vs ≤ High school	1.62	0.91	2.89			1.35	0.45	4.06			1.43	0.67	3.05		
	Non-university certificate, diploma vs ≤ High school	1.25	0.75	2.07			1.11	0.55	2.27			2.07	0.88	4.85		
	University certificate below bachelor’s ≤ High school	2.32	0.85	6.34			1.87	0.42	8.26			3.84	0.57	25.73		
	Bachelor’s degree vs ≤ High school	1.06	0.62	1.79			0.99	0.42	2.30			0.949	0.438	2.056		
	University degree, certificate above bachelor’s degree vs ≤ High school	1.62	0.85	3.09			1.83	0.57	5.88			2.10	0.87	5.04		
	Other vs High school or less	1.82	0.49	6.70			2.06	0.30	14.34			1.20	0.22	6.52		
**Marital Status**	Single vs Married/ common-law	1.39	0.69	2.80	1.42 (4, 18663)	0.23	2.03	0.70	5.91	**2.64** (4, 18663)	**0.03**	0.99	0.38	2.59	0.24 (4, 18663)	0.91
	Widowed vs Married/ common-law	1.53	0.94	2.50			**2.65**	**1.28**	**5.47**			1.32	0.57	3.03		
	Divorced vs Married/ common-law	0.83	0.46	1.51			0.85	0.37	1.93			0.90	0.30	2.69		
	Separated vs Married/ common-law	0.70	0.25	1.95			0.85	0.18	4.00			0.61	0.13	2.84		
**Rural**	Rural vs Urban	1.13	0.73	1.77	0.31 (1, 18666)	0.58	1.39	0.63	3.09	0.66 (1, 18666)	0.42	0.91	0.47	1.76	0.09 (1, 18666)	0.77

**Bold** values = statistically significant at p≤ 0.05

df num = degrees of freedom numerator, df den = degrees of freedom denominator

Of the 855 people with stroke in the final model, community ambulation was expressed as follows: 349 (41%) never or seldom walked outside their home or yard (never, 1-2days/week); 506 (59%) walked outside their home or yard sometimes or often (3–4 days/ week, 5–7 days/week). In the final model, variables associated with decreased odds of walking outdoors sometimes/often versus never/seldom were: being unable to walk 2–3 blocks and having difficulty walking 2–3 blocks (decreased endurance) compared to no difficulty. See Table 3. Factors associated with increased odds of walking outdoors sometimes/often versus never/seldom were: being in the 55–64 year age group and walking in better weather months (April-May, September). The S1 Table summarizes the variables that were statistically significant in the final models.

**Table 3:** Community ambulation—Final model.

		All participants with stroke n = 855					Females with Stroke n = 388					Males with Stroke n = 466				
Variables	Descriptor	Odds Ratio	95% CI		F Value (df num, df den)	p	Odds Ratio	95% CI		F Value (df num, df den)	p	Odds Ratio	95% CI		F Value (df num, df den)	p
**Endurance (Walk 2–3 blocks)**	Unable vs no difficulty	**0.12**	**0.05**	**0.27**	**22.40** (2, 18765)	**< .0001**	**0.13**	**0.04**	**0.42**	**15.46** (2, 18765)	**< .0001**	**0.03**	**0.01**	**0.19**	**10.16** (2, 18765)	**< .0001**
	Difficulty vs no difficulty	**0.34**	**0.22**	**0.51**			**0.20**	**0.10**	**0.37**			**0.38**	**0.20**	**0.70**		
**Pain**	Severe vs pain- free	0.68	0.39	1.18	2.52 (3, 18674)	0.06	0.43	0.17	1.10	**3.29** (3, 18674)	**0.02**	1.29	0.54	3.07	1.15 (3, 18674)	0.3
	Moderate vs pain- free	1.19	0.80	1.78			0.92	0.50	1.70			1.78	0.96	3.30		
	Mild vs pain- free	1.66	0.94	2.84			2.13	1.00	4.54			1.02	0.47	2.24		
**Depression**	All of the time (5-7days) vs Rarely or never (less than 1 day)						0.95	0.30	3.04	0.50	0.68	0.38	0.12	1.13	**4.14**	**0.01**
	Occasionally (3–4 days) vs Rarely or never (less than 1 day)						1.60	0.68	3.79			1.064	0.39	2.92		
	Some of the time (1–2 days) vs Rarely or never (less than 1 day)						1.36	0.65	2.86			**0.34**	**0.18**	**0.66**		
**Weather (month)**	February vs January	2.30	0.91	5.78	1.75 (11, 18756)	0.06	2.65	0.67	10.54	1.63 (11, 18756)	0.08	2.22	0.57	8.59	1.55 (11, 18756)	0.1
	March vs January	1.82	0.68	4.86			2.07	0.45	9.69			1.79	0.46	6.95		
	April vs January	**2.92**	**1.18**	**7.25**			2.90	0.70	12.06			2.26	0.65	7.94		
	May vs January	**3.05**	**1.38**	**6.72**			**3.77**	**1.12**	**12.73**			2.60	0.91	7.40		
	June vs January	2.05	01.0	4.26			**4.63**	**1.49**	**14.41**			0.91	0.34	2.45		
	July vs January	2.01	0.90	4.48			**5.80**	**1.75**	**19.22**			0.88	0.28	2.79		
	August vs January	1.94	0.83	4.52			3.20	0.96	10.72			1.37	0.38	4.90		
	September vs January	**3.35**	**1.30**	**8.65**			**7.29**	**2.01**	**26.47**			2.33	0.61	8.95		
	October vs January	2.60	0.93	7.29			**10.88**	**1.93**	**61.51**			1.18	0.34	4.02		
	November vs January	1.38	0.59	3.22			**3.86**	**1.11**	**13.40**			0.79	0.28	2.28		
	December vs January	1.04	0.48	2.25			1.65	0.51	5.31			0.60	0.22	1.69		
**Age group**	45–54 vs 75–85	1.14	0.61	2.14	2.15 (3, 18764)	0.09	0.81	0.27	2.43	2.09 (3, 18764)	0.10	1.36	0.58	3.16	0.54 (3, 18764)	0.66
	55–64 vs 75–85	**1.83**	**1.14**	**2.94**			**2.32**	**1.14**	**4.73**			1.50	0.76	2.98		
	65–74 vs 75–85	1.27	0.86	1.88			1.20	0.62	2.33			1.05	0.58	1.87		
**Sex**	Female vs Male	0.74	0.51	1.08	2.49 (1, 18766)	0.11										
**Income**	>$20,000, < $50,000 vs <20,000	0.77	0.43	1.37	0.65 (5, 18762)	0.66	0.81	0.37	1.79	1.05 (5, 18762)	0.39	0.61	0.18	2.01	0.65 (5, 18762)	0.66
	≥$50,000, < $100,000 vs < $20,000	0.75	0.39	1.44			1.26	0.47	3.33			0.44	0.13	1.52		
	≥$100,000, < $150,000 vs < $20,000	0.67	0.29	1.58			0.33	0.07	1.66			0.57	0.14	2.36		
	$150,000 + vs < $20,000	1.46	0.48	4.47			1.37	0.25	7.45			0.82	0.13	5.06		
	No response vs < $20,000	0.97	0.44	2.13			1.35	0.49	3.77			0.43	0.10	1.90		
**Education**	No post-secondary degree, certificate or diploma vs ≤ High school	0.96	0.49	1.86	0.83 (7, 18760)	0.56	1.48	0.53	4.18	0.48 (7, 18760)	0.85	0.79	0.32	1.97	0.79 (7, 18760)	0.60
	Trade certificate, diploma or apprenticeship vs ≤ High school	1.53	0.87	2.69			1.26	0.39	4.03			1.37	0.67	2.82		
	Non-university certificate, diploma vs ≤ High school	1.22	0.73	2.02			1.15	0.56	2.36			1.72	0.75	3.96		
	University certificate below bachelor’s ≤ High school	2.14	0.84	5.47			2.14	0.54	8.46			2.83	0.54	14.76		
	Bachelor’s degree vs ≤ High school	1.09	0.65	1.81			0.96	0.43	2.18			0.95	0.45	1.97		
	University degree, certificate above bachelor’s degree vs ≤ High school	1.49	0.80	2.71			1.96	0.66	5.85			1.67	0.74	3.79		
	Other vs High school or less	1.80	0.55	5.91			1.92	0.24	15.27			1.37	0.27	6.99		
**Marital Status**	Single vs Married/ common-law	1.39	0.70	2.76	1.23 (4, 18763)	0.30	1.66	0.58	4.75	2.04 (4, 18763)	0.08	0.99	0.38	2.63	0.01 (4, 18763)	0.10
	Widowed vs Married/ common-law	1.46	0.90	2.37			**2.25**	**1.14**	**4.44**			1.05	0.47	2.35		
	Divorced vs Married/ common-law	0.80	0.45	1.42			0.76	0.33	1.74			1.02	0.35	3.00		
	Separated vs Married/ common-law	0.83	0.30	2.27			0.88	0.18	4.39			0.88	0.19	4.17		
**Rural**	Rural vs Urban	1.18	0.77	1.82	0.59 (1, 18766)	0.44	1.26	0.61	2.59	0.39 (1, 18766)	0.53	0.10	0.52	1.90	0.00 (1, 18766)	0.98

**Bold** values = statistically significant at p≤ 0.05

df num = degrees of freedom numerator, df den = degrees of freedom denominator

For the sex-specific final models, females were less likely to walk outdoors sometimes/often versus never/seldom: if they were unable or having difficulty walking 2–3 blocks (decreased endurance) compared to having no difficulty. They were more likely to walk outdoors sometimes/often versus never/seldom in better weather months (May-July, September-November) versus January, if they were aged 55–64 versus 75–85 years, or were widowed versus being married or common-law.

For males, factors related to decreased likelihood of walking outdoors sometimes/often versus never/seldom included being unable or having difficulty walking 2–3 blocks (decreased endurance) and having depression sometimes versus rarely or never. Good weather months did not appear to have an association with outdoor walking.

## Discussion

This study is unique from other studies evaluating community ambulation post stroke. Using CLSA data, we were able to evaluate a large Canadian population-based sample of people living with stroke, aged 45–85, and evaluate a substantial number of self-report variables. Other studies have primarily looked at physical assessment variables. Future studies could use follow-up times from CLSA to look at changes over time in community ambulation and the self-reported variables linked to community ambulation. The use of self-reported variables in evaluating community ambulation post stroke could be beneficial for future large studies or when in-person follow-up is not possible.

In this study, 59% of 855 people with stroke walked outside their home or yard sometimes or often (3–4 days/week, 5–7 days/week). Data regarding time since stroke was not available. Other studies have suggested different values, using different methods of determining community ambulation, at varying times post stroke. One study found that between 1 and 3 years post stroke, 57.5% of 40 participants were “independent community walkers” while 35% were “limited community walkers” [12]. In another study, 37% of 30 participants at three months post stroke were independent community ambulators [8].

An important factor associated with community ambulation was endurance. Low endurance (being unable to walk or having difficulty walking the distance of 2–3 blocks) was associated with less frequent community ambulation. Addressing endurance after stroke (with an initial focus on the 2–3 block distance) in rehabilitation assessment, intervention, and goal setting may assist in people attaining higher levels of community ambulation. In a previous study, the six-minute walk test, a measure of endurance, predicted community ambulation after stroke for people who were high functioning [14].

People with stroke find walking in the community challenging due to unpredictability in the environment [29, 30]. It is important to understand a variety of factors which may be associated with the frequency of community ambulation for people with stroke. Good weather months were associated with increased odds of community ambulation. Poor weather (very cold or very hot) is known to be a barrier to walking outdoors after stroke [6]. Research and programs that enable people with stroke to maintain walking ability all year should be a priority. Some people with stroke may stay active and participate in winter months, but also identify challenges with winter weather conditions that limit activities [31].

The number of falls that occurred in the previous year was not associated with community ambulation. It has been previously noted that a history of falls did not affect outdoor walking activity in people with stroke [12]. We did not identify an association between community ambulation post stroke and self-rated health, though this has been identified in another study [18] that used different measures of self-rated health. Younger age has been associated with being able to ambulate in the community after stroke [17], as we also found.

Sex was not associated with community ambulation in the full model, consistent with previous findings [17]. However, other than being able to walk a distance of 2–3 blocks (endurance), models specific to females and males estimated different factors that were associated with walking outdoors in the community. We found that males with stroke were less likely to walk outdoors if they sometimes had depression. Similarly, in a previous study, individuals with stroke were more likely to be able to walk in the community if they did not have depression; 53% of the people in that study were male [17]. Women were more likely to walk outdoors in better weather months, but this was not the case for males in this study. A qualitative study found that bad weather was a barrier to outdoor walking for people with stroke; 42% of the participants were female [6].

### Limitations

A limitation of this study is that we do not know the severity of the stroke, affected side, type of stroke, or when the stroke occurred as this information was not part of the data collection in the CLSA tracking questionnaire. We also did not know an individual’s community ambulation level prior to stroke. This information may have helped to clarify some of our findings. This study included people with stroke living in the community with an average age of 68 (age 45–85), so it may not be generalizable to other age groups. This dataset included only self-report measures; observed or clinician evaluated physical factors which may be associated with community ambulation were not part of the Tracking Main Wave dataset. However, this can also be viewed as a positive aspect, for future large studies of stroke or remote / virtual evaluations where physical assessment may not be possible and self-report is the method of choice.

## Conclusion

Limited walking endurance (having difficulty walking or being unable to walk the distance of 2–3 blocks) is a factor associated with decreased odds of people with stroke walking in the community; this factor can be addressed clinically and in community exercise programs. Seasonal variability in community ambulation needs to be considered to address or prevent functional decline in ambulation over time. These results can be used by rehabilitation clinicians, community programs, policy makers and researchers to assist in choosing, designing and evaluating intervention programs to promote community ambulation for people with stroke.

## Supporting information

S1 Table Community ambulation—Summary of results. OR = odds ratio, 95% CI = 95% confidence interval.
(TIF)
