# Correction: Self-reported factors associated with community ambulation after stroke: The Canadian Longitudinal Study on Aging

**DOI:** 10.1371/journal.pone.0308733

**Published:** 2024-08-08

**Authors:** Ruth Barclay, Sandra C. Webber, Jacquie Ripat, Scott Nowicki, Robert Tate

The following information is missing from the Funding statement: Funding for the Canadian Longitudinal Study on Aging (CLSA) is provided by the Government of Canada through the Canadian Institutes of Health Research (CIHR) under grant reference: LSA 94473, and the Canada Foundation for Innovation, as well as the following provinces: Newfoundland, Nova Scotia, Quebec, Ontario, Manitoba, Alberta, and British Columbia.

The correct funding statement is: RB, SCW, and JR received funding for this study from the Endowment Fund of the College of Rehabilitation Sciences at the University of Manitoba. Funding for the Canadian Longitudinal Study on Aging (CLSA) is provided by the Government of Canada through the Canadian Institutes of Health Research (CIHR) under grant reference: LSA 94473, and the Canada Foundation for Innovation, as well as the following provinces: Newfoundland, Nova Scotia, Quebec, Ontario, Manitoba, Alberta, and British Columbia. The funders had no role in study design, data collection and analysis, decision to publish, or preparation of the manuscript.
