# Supplementary figures and images for: Self-reported factors associated with community ambulation after stroke: The Canadian Longitudinal Study on Aging
Source: PLoS One. 2024 Mar 28;19(3):e0299569. doi: 10.1371/journal.pone.0299569 (PMC10977696; doi:10.1371/journal.pone.0299569)

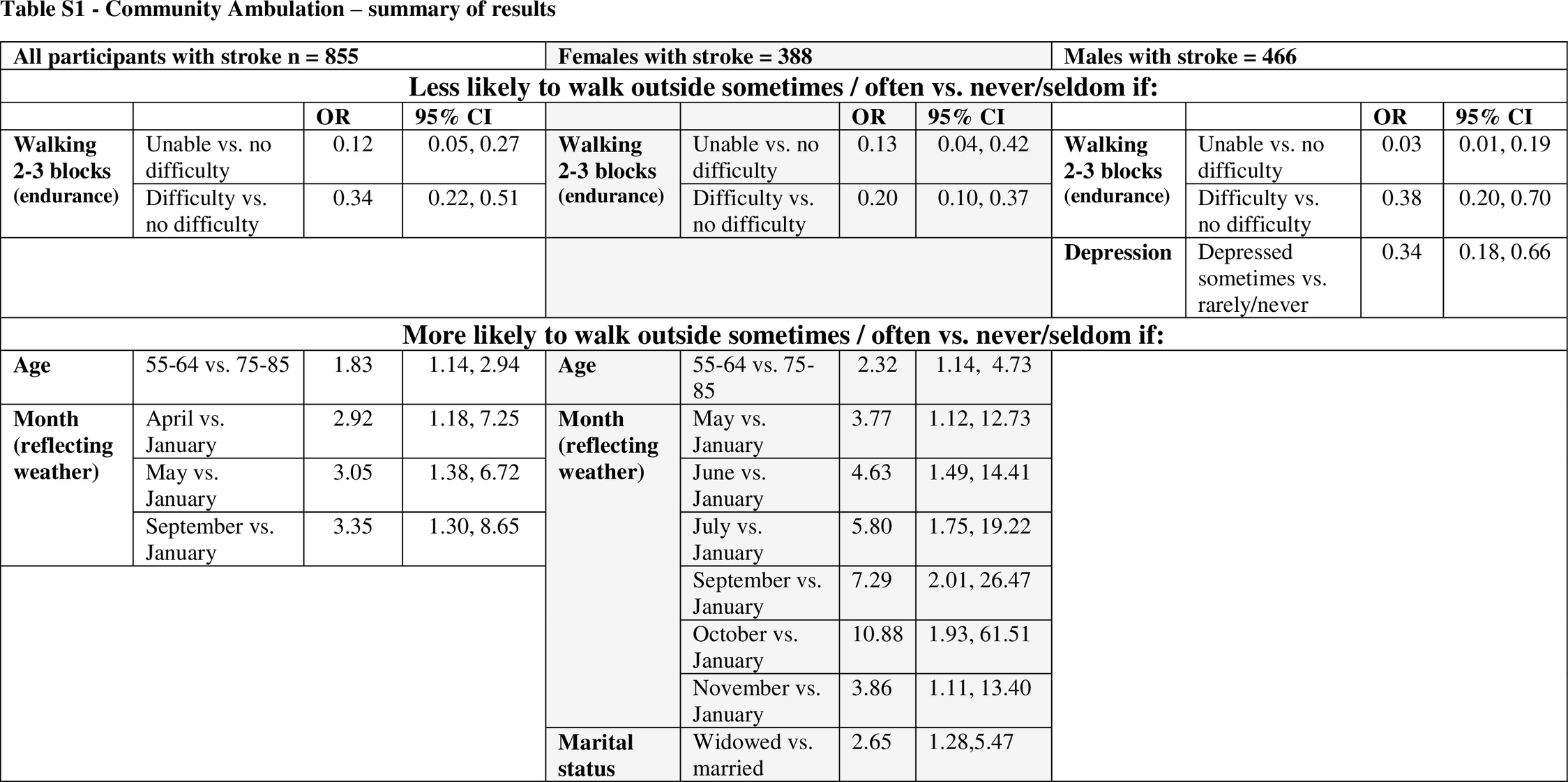

Supplement: S1 Table — OR = odds ratio, 95% CI = 95% confidence interval. (TIF) [file pone.0299569.s001.tif]
